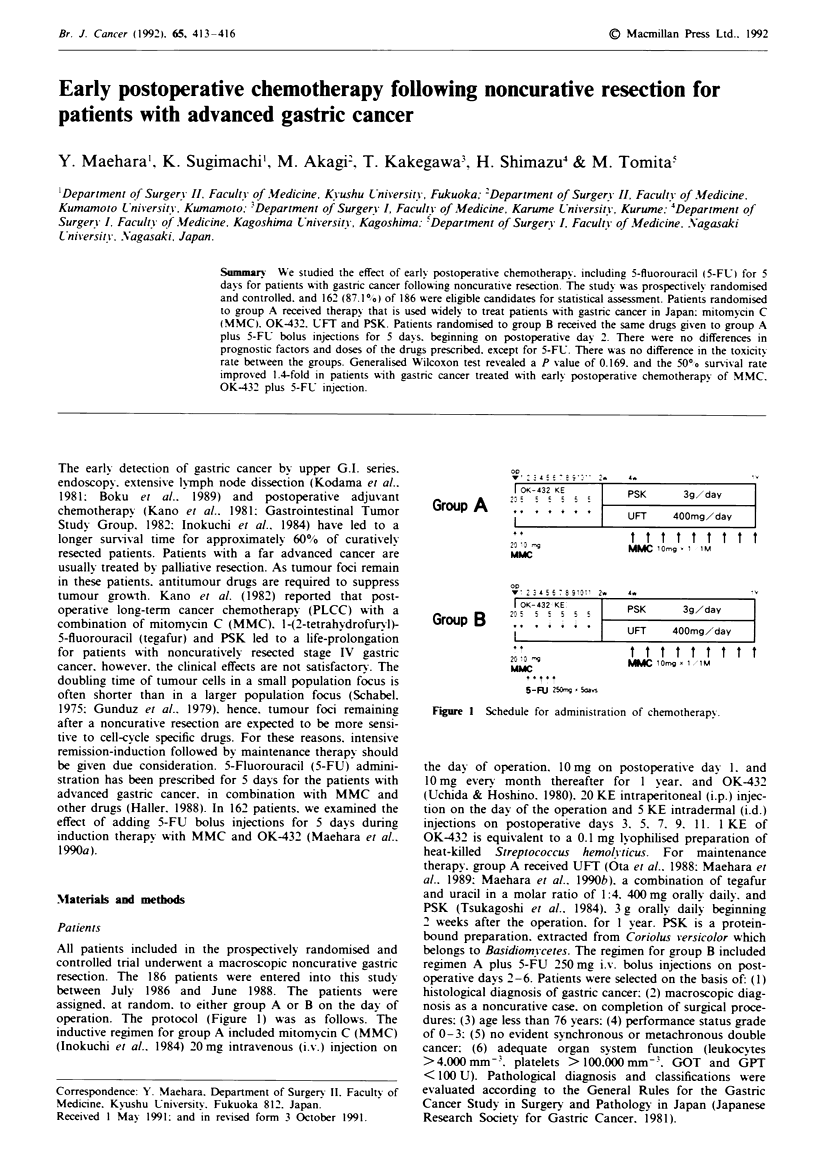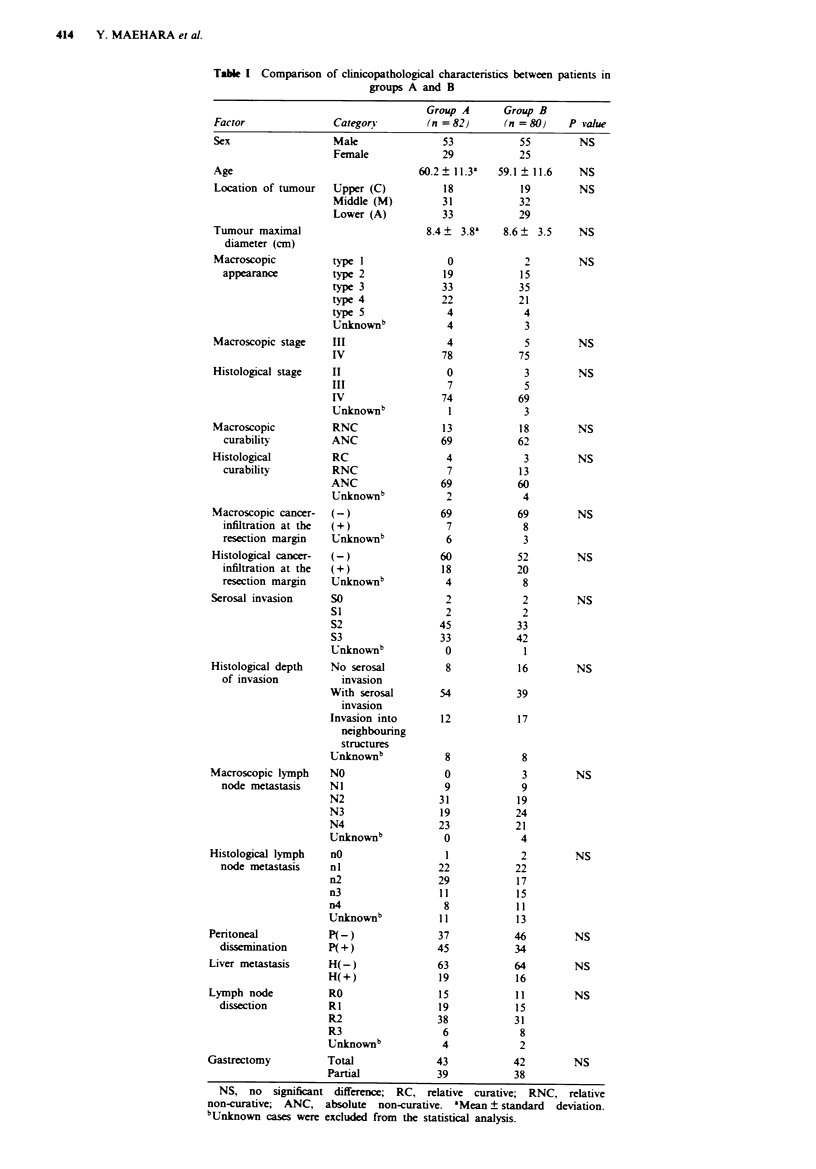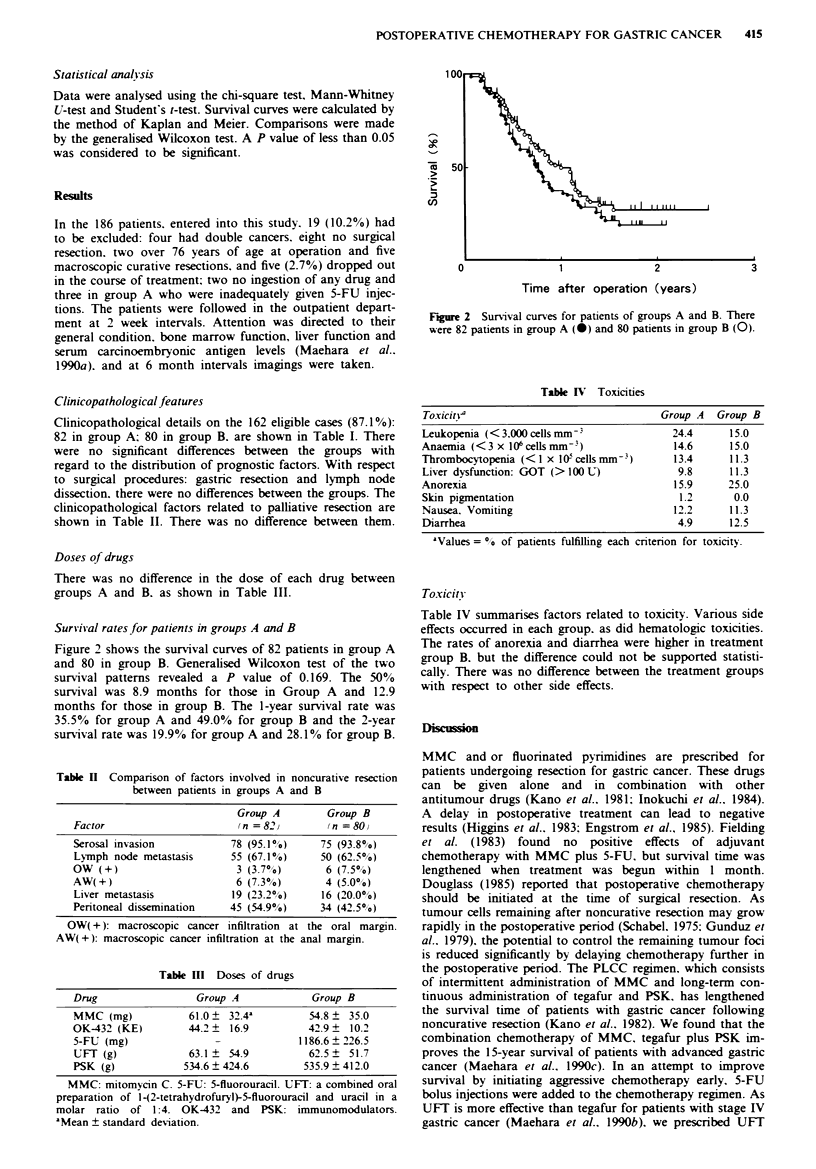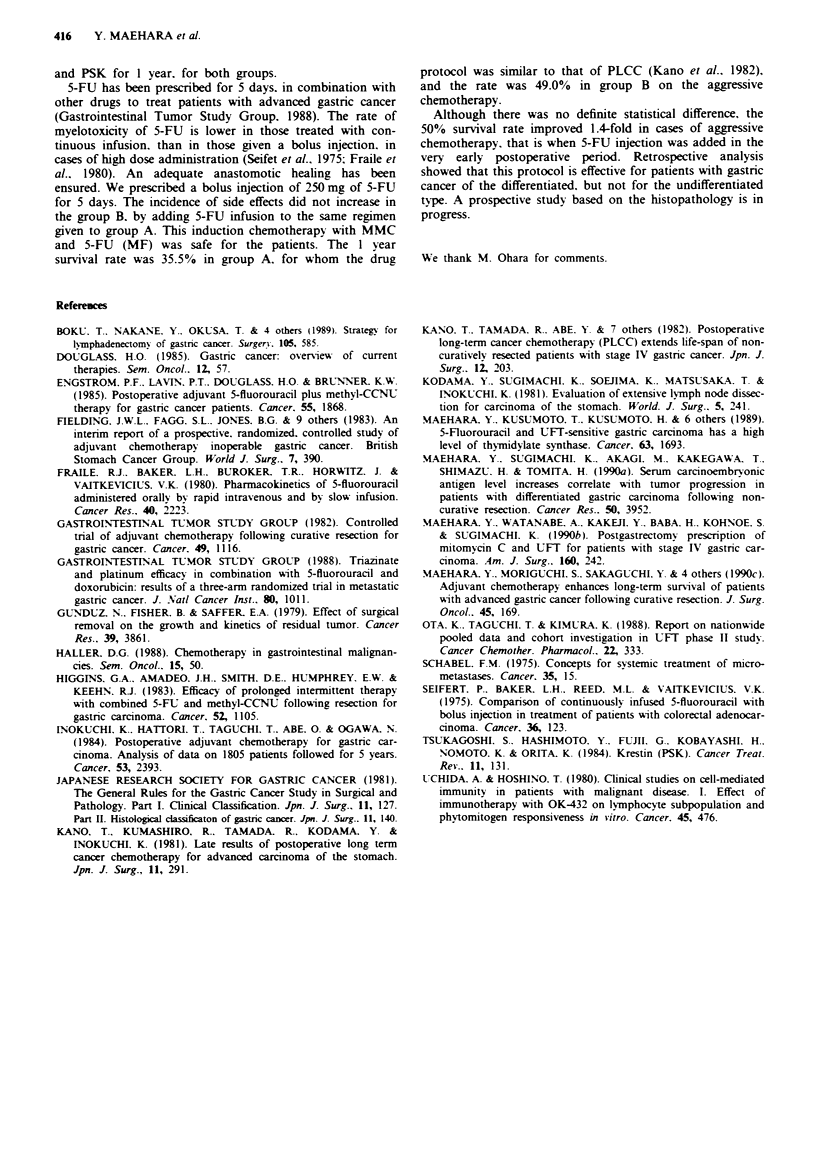# Early postoperative chemotherapy following noncurative resection for patients with advanced gastric cancer.

**DOI:** 10.1038/bjc.1992.84

**Published:** 1992-03

**Authors:** Y. Maehara, K. Sugimachi, M. Akagi, T. Kakegawa, H. Shimazu, M. Tomita

**Affiliations:** Department of Surgery II, Faculty of Medicine, Kyushu University, Fukuoka, Japan.

## Abstract

We studied the effect of early postoperative chemotherapy, including 5-fluorouracil (5-FU) for 5 days for patients with gastric cancer following noncurative resection. The study was prospectively randomised and controlled, and 162 (87.1%) of 186 were eligible candidates for statistical assessment. Patients randomised to group A received therapy that is used widely to treat patients with gastric cancer in Japan; mitomycin C (MMC), OK-432, UFT and PSK. Patients randomised to group B received the same drugs given to group A plus 5-FU bolus injections for 5 days, beginning on postoperative day 2. There were no differences in prognostic factors and doses of the drugs prescribed, except for 5-FU. There was no difference in the toxicity rate between the groups. Generalised Wilcoxon test revealed a P value of 0.169, and the 50% survival rate improved 1.4-fold in patients with gastric cancer treated with early postoperative chemotherapy of MMC, OK-432 plus 5-FU injection.


*Br. J. Cancer* (1992), **65**, 413–416 © Macmillan Press Ltd., 1992

### Early postoperative chemotherapy following noncurative resection for patients with advanced gastric cancer

Y. Maehara[1], K. Sugimachi[1], M. Akagi[2], T. Kakegawa[3], H. Shimazu[4] & M. Tomita[5]

[1]*Department of Surgery II, Faculty of Medicine, Kyushu University, Fukuoka;* [2]*Department of Surgery II, Faculty of Medicine, Kumamoto University, Kumamoto;* [3]*Department of Surgery I, Faculty of Medicine, Karume University, Kurume;* [4]*Department of Surgery I, Faculty of Medicine, Kagoshima University, Kagoshima;* [5]*Department of Surgery I, Faculty of Medicine, Nagasaki University, Nagasaki, Japan.*

**Summary** We studied the effect of early postoperative chemotherapy, including 5-fluorouracil (5-FU) for 5 days for patients with gastric cancer following noncurative resection. The study was prospectively randomised and controlled, and 162 (87.1%) of 186 were eligible candidates for statistical assessment. Patients randomised to group A received therapy that is used widely to treat patients with gastric cancer in Japan: mitomycin C (MMC), OK-432, UFT and PSK. Patients randomised to group B received the same drugs given to group A plus 5-FU bolus injections for 5 days, beginning on postoperative day 2. There were no differences in prognostic factors and doses of the drugs prescribed, except for 5-FU. There was no difference in the toxicity rate between the groups. Generalised Wilcoxon test revealed a *P* value of 0.169, and the 50% survival rate improved 1.4-fold in patients with gastric cancer treated with early postoperative chemotherapy of MMC, OK-432 plus 5-FU injection.

The early detection of gastric cancer by upper G.I. series, endoscopy, extensive lymph node dissection (Kodama *et al.*, 1981; Boku *et al.*, 1989) and postoperative adjuvant chemotherapy (Kano *et al.*, 1981; Gastrointestinal Tumor Study Group, 1982; Inokuchi *et al.*, 1984) have led to a longer survival time for approximately 60% of curatively resected patients. Patients with a far advanced cancer are usually treated by palliative resection. As tumour foci remain in these patients, antitumour drugs are required to suppress tumour growth. Kano *et al.* (1982) reported that post-operative long-term cancer chemotherapy (PLCC) with a combination of mitomycin C (MMC), 1-(2-tetrahydrofuryl)-5-fluorouracil (tegafur) and PSK led to a life-prolongation for patients with noncuratively resected stage IV gastric cancer, however, the clinical effects are not satisfactory. The doubling time of tumour cells in a small population focus is often shorter than in a larger population focus (Schabel, 1975; Gunduz *et al.*, 1979), hence, tumour foci remaining after a noncurative resection are expected to be more sensitive to cell-cycle specific drugs. For these reasons, intensive remission-induction followed by maintenance therapy should be given due consideration. 5-Fluorouracil (5-FU) administration has been prescribed for 5 days for the patients with advanced gastric cancer, in combination with MMC and other drugs (Haller, 1988). In 162 patients, we examined the effect of adding 5-FU bolus injections for 5 days during induction therapy with MMC and OK-432 (Maehara *et al.*, 1990*a*).

**Figure 1** Schedule for administration of chemotherapy.

### Materials and methods

### *Patients*

All patients included in the prospectively randomised and controlled trial underwent a macroscopic noncurative gastric resection. The 186 patients were entered into this study between July 1986 and June 1988. The patients were assigned, at random, to either group A or B on the day of operation. The protocol (Figure 1) was as follows. The inductive regimen for group A included mitomycin C (MMC) (Inokuchi *et al.*, 1984) 20 mg intravenous (i.v.) injection on the day of operation, 10 mg on postoperative day 1, and 10 mg every month thereafter for 1 year, and OK-432 (Uchida & Hoshino, 1980), 20 KE intraperitoneal (i.p.) injection on the day of the operation and 5 KE intradermal (i.d.) injections on postoperative days 3, 5, 7, 9, 11. 1 KE of OK-432 is equivalent to a 0.1 mg lyophilised preparation of heat-killed *Streptococcus hemolyticus*. For maintenance therapy, group A received UFT (Ota *et al.*, 1988; Maehara *et al.*, 1989; Maehara *et al.*, 1990*b*), a combination of tegafur and uracil in a molar ratio of 1:4, 400 mg orally daily, and PSK (Tsukagoshi *et al.*, 1984), 3 g orally daily beginning 2 weeks after the operation, for 1 year. PSK is a protein-bound preparation, extracted from *Coriolus versicolor* which belongs to *Basidiomycetes*. The regimen for group B included regimen A plus 5-FU 250 mg i.v. bolus injections on post-operative days 2–6. Patients were selected on the basis of: (1) histological diagnosis of gastric cancer; (2) macroscopic diagnosis as a noncurative case, on completion of surgical procedures; (3) age less than 76 years; (4) performance status grade of 0–3; (5) no evident synchronous or metachronous double cancer; (6) adequate organ system function (leukocytes $>4,000\ mm^{-3}$, platelets $>100,000\ mm^{-3}$, GOT and GPT $<100$ U). Pathological diagnosis and classifications were evaluated according to the General Rules for the Gastric Cancer Study in Surgery and Pathology in Japan (Japanese Research Society for Gastric Cancer, 1981).

Correspondence: Y. Maehara, Department of Surgery II, Faculty of Medicine, Kyushu University, Fukuoka 812, Japan.
Received 1 May 1991; and in revised form 3 October 1991.

**Table I** Comparison of clinicopathological characteristics between patients in groups A and B

| Factor | Category | Group A (n = 82) | Group B (n = 80) | P value |
|---|---|---|---|---|
| Sex | Male | 53 | 55 | NS |
| | Female | 29 | 25 | |
| Age | | 60.2 ± 11.3[a] | 59.1 ± 11.6 | NS |
| Location of tumour | Upper (C) | 18 | 19 | NS |
| | Middle (M) | 31 | 32 | |
| | Lower (A) | 33 | 29 | |
| Tumour maximal diameter (cm) | | 8.4 ± 3.8[a] | 8.6 ± 3.5 | NS |
| Macroscopic appearance | type 1 | 0 | 2 | NS |
| | type 2 | 19 | 15 | |
| | type 3 | 33 | 35 | |
| | type 4 | 22 | 21 | |
| | type 5 | 4 | 4 | |
| | Unknown[b] | 4 | 3 | |
| Macroscopic stage | III | 4 | 5 | NS |
| | IV | 78 | 75 | |
| Histological stage | II | 0 | 3 | NS |
| | III | 7 | 5 | |
| | IV | 74 | 69 | |
| | Unknown[b] | 1 | 3 | |
| Macroscopic curability | RNC | 13 | 18 | NS |
| | ANC | 69 | 62 | |
| Histological curability | RC | 4 | 3 | NS |
| | RNC | 7 | 13 | |
| | ANC | 69 | 60 | |
| | Unknown[b] | 2 | 4 | |
| Macroscopic cancer-infiltration at the resection margin | (−) | 69 | 69 | NS |
| | (+) | 7 | 8 | |
| | Unknown[b] | 6 | 3 | |
| Histological cancer-infiltration at the resection margin | (−) | 60 | 52 | NS |
| | (+) | 18 | 20 | |
| | Unknown[b] | 4 | 8 | |
| Serosal invasion | S0 | 2 | 2 | NS |
| | S1 | 2 | 2 | |
| | S2 | 45 | 33 | |
| | S3 | 33 | 42 | |
| | Unknown[b] | 0 | 1 | |
| Histological depth of invasion | No serosal invasion | 8 | 16 | NS |
| | With serosal invasion | 54 | 39 | |
| | Invasion into neighbouring structures | 12 | 17 | |
| | Unknown[b] | 8 | 8 | |
| Macroscopic lymph node metastasis | N0 | 0 | 3 | NS |
| | N1 | 9 | 9 | |
| | N2 | 31 | 19 | |
| | N3 | 19 | 24 | |
| | N4 | 23 | 21 | |
| | Unknown[b] | 0 | 4 | |
| Histological lymph node metastasis | n0 | 1 | 2 | NS |
| | n1 | 22 | 22 | |
| | n2 | 29 | 17 | |
| | n3 | 11 | 15 | |
| | n4 | 8 | 11 | |
| | Unknown[b] | 11 | 13 | |
| Peritoneal dissemination | P(−) | 37 | 46 | NS |
| | P(+) | 45 | 34 | |
| Liver metastasis | H(−) | 63 | 64 | NS |
| | H(+) | 19 | 16 | |
| Lymph node dissection | R0 | 15 | 11 | NS |
| | R1 | 19 | 15 | |
| | R2 | 38 | 31 | |
| | R3 | 6 | 8 | |
| | Unknown[b] | 4 | 2 | |
| Gastrectomy | Total | 43 | 42 | NS |
| | Partial | 39 | 38 | |

NS, no significant difference; RC, relative curative; RNC, relative non-curative; ANC, absolute non-curative. [a]Mean ± standard deviation. [b]Unknown cases were excluded from the statistical analysis.

*Statistical analysis*

Data were analysed using the chi-square test, Mann-Whitney *U*-test and Student's *t*-test. Survival curves were calculated by the method of Kaplan and Meier. Comparisons were made by the generalised Wilcoxon test. A *P* value of less than 0.05 was considered to be significant.

### Results

In the 186 patients, entered into this study, 19 (10.2%) had to be excluded: four had double cancers, eight no surgical resection, two over 76 years of age at operation and five macroscopic curative resections, and five (2.7%) dropped out in the course of treatment; two no ingestion of any drug and three in group A who were inadequately given 5-FU injections. The patients were followed in the outpatient department at 2 week intervals. Attention was directed to their general condition, bone marrow function, liver function and serum carcinoembryonic antigen levels (Maehara *et al*., 1990*a*), and at 6 month intervals imagings were taken.

*Clinicopathological features*

Clinicopathological details on the 162 eligible cases (87.1%): 82 in group A; 80 in group B, are shown in Table I. There were no significant differences between the groups with regard to the distribution of prognostic factors. With respect to surgical procedures: gastric resection and lymph node dissection, there were no differences between the groups. The clinicopathological factors related to palliative resection are shown in Table II. There was no difference between them.

*Doses of drugs*

There was no difference in the dose of each drug between groups A and B, as shown in Table III.

*Survival rates for patients in groups A and B*

Figure 2 shows the survival curves of 82 patients in group A and 80 in group B. Generalised Wilcoxon test of the two survival patterns revealed a *P* value of 0.169. The 50% survival was 8.9 months for those in Group A and 12.9 months for those in group B. The 1-year survival rate was 35.5% for group A and 49.0% for group B and the 2-year survival rate was 19.9% for group A and 28.1% for group B.

**Table II** Comparison of factors involved in noncurative resection between patients in groups A and B

| Factor | Group A (n = 82) | Group B (n = 80) |
|---|---|---|
| Serosal invasion | 78 (95.1%) | 75 (93.8%) |
| Lymph node metastasis | 55 (67.1%) | 50 (62.5%) |
| OW (+) | 3 (3.7%) | 6 (7.5%) |
| AW(+) | 6 (7.3%) | 4 (5.0%) |
| Liver metastasis | 19 (23.2%) | 16 (20.0%) |
| Peritoneal dissemination | 45 (54.9%) | 34 (42.5%) |

OW(+): macroscopic cancer infiltration at the oral margin. AW(+): macroscopic cancer infiltration at the anal margin.

**Table III** Doses of drugs

| Drug | Group A | Group B |
|---|---|---|
| MMC (mg) | 61.0 ± 32.4[a] | 54.8 ± 35.0 |
| OK-432 (KE) | 44.2 ± 16.9 | 42.9 ± 10.2 |
| 5-FU (mg) | - | 1186.6 ± 226.5 |
| UFT (g) | 63.1 ± 54.9 | 62.5 ± 51.7 |
| PSK (g) | 534.6 ± 424.6 | 535.9 ± 412.0 |

MMC: mitomycin C. 5-FU: 5-fluorouracil. UFT: a combined oral preparation of 1-(2-tetrahydrofuryl)-5-fluorouracil and uracil in a molar ratio of 1:4. OK-432 and PSK: immunomodulators. [a]Mean ± standard deviation.

**Figure 2** Survival curves for patients of groups A and B. There were 82 patients in group A (●) and 80 patients in group B (○).

**Table IV** Toxicities

| Toxicity[a] | Group A | Group B |
|---|---|---|
| Leukopenia (< 3,000 cells mm$^{-3}$) | 24.4 | 15.0 |
| Anaemia (< 3 × 10$^{6}$ cells mm$^{-3}$) | 14.6 | 15.0 |
| Thrombocytopenia (< 1 × 10$^{5}$ cells mm$^{-3}$) | 13.4 | 11.3 |
| Liver dysfunction: GOT (> 100 U) | 9.8 | 11.3 |
| Anorexia | 15.9 | 25.0 |
| Skin pigmentation | 1.2 | 0.0 |
| Nausea, Vomiting | 12.2 | 11.3 |
| Diarrhea | 4.9 | 12.5 |

[a]Values = % of patients fulfilling each criterion for toxicity.

*Toxicity*

Table IV summarises factors related to toxicity. Various side effects occurred in each group, as did hematologic toxicities. The rates of anorexia and diarrhea were higher in treatment group B, but the difference could not be supported statistically. There was no difference between the treatment groups with respect to other side effects.

### Discussion

MMC and or fluorinated pyrimidines are prescribed for patients undergoing resection for gastric cancer. These drugs can be given alone and in combination with other antitumour drugs (Kano *et al*., 1981; Inokuchi *et al*., 1984). A delay in postoperative treatment can lead to negative results (Higgins *et al*., 1983; Engstrom *et al*., 1985). Fielding *et al*. (1983) found no positive effects of adjuvant chemotherapy with MMC plus 5-FU, but survival time was lengthened when treatment was begun within 1 month. Douglass (1985) reported that postoperative chemotherapy should be initiated at the time of surgical resection. As tumour cells remaining after noncurative resection may grow rapidly in the postoperative period (Schabel, 1975; Gunduz *et al*., 1979), the potential to control the remaining tumour foci is reduced significantly by delaying chemotherapy further in the postoperative period. The PLCC regimen, which consists of intermittent administration of MMC and long-term continuous administration of tegafur and PSK, has lengthened the survival time of patients with gastric cancer following noncurative resection (Kano *et al*., 1982). We found that the combination chemotherapy of MMC, tegafur plus PSK improves the 15-year survival of patients with advanced gastric cancer (Maehara *et al*., 1990*c*). In an attempt to improve survival by initiating aggressive chemotherapy early, 5-FU bolus injections were added to the chemotherapy regimen. As UFT is more effective than tegafur for patients with stage IV gastric cancer (Maehara *et al*., 1990*b*), we prescribed UFT

and PSK for 1 year, for both groups.

5-FU has been prescribed for 5 days, in combination with other drugs to treat patients with advanced gastric cancer (Gastrointestinal Tumor Study Group, 1988). The rate of myelotoxicity of 5-FU is lower in those treated with continuous infusion, than in those given a bolus injection, in cases of high dose administration (Seifet *et al.*, 1975; Fraile *et al.*, 1980). An adequate anastomotic healing has been ensured. We prescribed a bolus injection of 250 mg of 5-FU for 5 days. The incidence of side effects did not increase in the group B, by adding 5-FU infusion to the same regimen given to group A. This induction chemotherapy with MMC and 5-FU (MF) was safe for the patients. The 1 year survival rate was 35.5% in group A, for whom the drug protocol was similar to that of PLCC (Kano *et al.*, 1982), and the rate was 49.0% in group B on the aggressive chemotherapy.

Although there was no definite statistical difference, the 50% survival rate improved 1.4-fold in cases of aggressive chemotherapy, that is when 5-FU injection was added in the very early postoperative period. Retrospective analysis showed that this protocol is effective for patients with gastric cancer of the differentiated, but not for the undifferentiated type. A prospective study based on the histopathology is in progress.

We thank M. Ohara for comments.

### References

BOKU, T., NAKANE, Y., OKUSA, T. & 4 others (1989). Strategy for lymphadenectomy of gastric cancer. *Surgery*, **105**, 585.

DOUGLASS, H.O. (1985). Gastric cancer: overview of current therapies. *Sem. Oncol.*, **12**, 57.

ENGSTROM, P.F., LAVIN, P.T., DOUGLASS, H.O. & BRUNNER, K.W. (1985). Postoperative adjuvant 5-fluorouracil plus methyl-CCNU therapy for gastric cancer patients. *Cancer*, **55**, 1868.

FIELDING, J.W.L., FAGG, S.L., JONES, B.G. & 9 others (1983). An interim report of a prospective, randomized, controlled study of adjuvant chemotherapy inoperable gastric cancer. British Stomach Cancer Group. *World J. Surg.*, **7**, 390.

FRAILE, R.J., BAKER, L.H., BUROKER, T.R., HORWITZ, J. & VAITKEVICIUS, V.K. (1980). Pharmacokinetics of 5-fluorouracil administered orally by rapid intravenous and by slow infusion. *Cancer Res.*, **40**, 2223.

GASTROINTESTINAL TUMOR STUDY GROUP (1982). Controlled trial of adjuvant chemotherapy following curative resection for gastric cancer. *Cancer*, **49**, 1116.

GASTROINTESTINAL TUMOR STUDY GROUP (1988). Triazinate and platinum efficacy in combination with 5-fluorouracil and doxorubicin: results of a three-arm randomized trial in metastatic gastric cancer. *J. Natl Cancer Inst.*, **80**, 1011.

GUNDUZ, N., FISHER, B. & SAFFER, E.A. (1979). Effect of surgical removal on the growth and kinetics of residual tumor. *Cancer Res.*, **39**, 3861.

HALLER, D.G. (1988). Chemotherapy in gastrointestinal malignancies. *Sem. Oncol.*, **15**, 50.

HIGGINS, G.A., AMADEO, J.H., SMITH, D.E., HUMPHREY, E.W. & KEEHN, R.J. (1983). Efficacy of prolonged intermittent therapy with combined 5-FU and methyl-CCNU following resection for gastric carcinoma. *Cancer*, **52**, 1105.

INOKUCHI, K., HATTORI, T., TAGUCHI, T., ABE, O. & OGAWA, N. (1984). Postoperative adjuvant chemotherapy for gastric carcinoma. Analysis of data on 1805 patients followed for 5 years. *Cancer*, **53**, 2393.

JAPANESE RESEARCH SOCIETY FOR GASTRIC CANCER (1981). The General Rules for the Gastric Cancer Study in Surgical and Pathology. Part I. Clinical Classification. *Jpn. J. Surg.*, **11**, 127. Part II. Histological classificaton of gastric cancer. *Jpn. J. Surg.*, **11**, 140.

KANO, T., KUMASHIRO, R., TAMADA, R., KODAMA, Y. & INOKUCHI, K. (1981). Late results of postoperative long term cancer chemotherapy for advanced carcinoma of the stomach. *Jpn. J. Surg.*, **11**, 291.

KANO, T., TAMADA, R., ABE, Y. & 7 others (1982). Postoperative long-term cancer chemotherapy (PLCC) extends life-span of non-curatively resected patients with stage IV gastric cancer. *Jpn. J. Surg.*, **12**, 203.

KODAMA, Y., SUGIMACHI, K., SOEJIMA, K., MATSUSAKA, T. & INOKUCHI, K. (1981). Evaluation of extensive lymph node dissection for carcinoma of the stomach. *World. J. Surg.*, **5**, 241.

MAEHARA, Y., KUSUMOTO, T., KUSUMOTO, H. & 6 others (1989). 5-Fluorouracil and UFT-sensitive gastric carcinoma has a high level of thymidylate synthase. *Cancer*, **63**, 1693.

MAEHARA, Y., SUGIMACHI, K., AKAGI, M., KAKEGAWA, T., SHIMAZU, H. & TOMITA, H. (1990*a*). Serum carcinoembryonic antigen level increases correlate with tumor progression in patients with differentiated gastric carcinoma following non-curative resection. *Cancer Res.*, **50**, 3952.

MAEHARA, Y., WATANABE, A., KAKEJI, Y., BABA, H., KOHNOE, S. & SUGIMACHI, K. (1990*b*). Postgastrectomy prescription of mitomycin C and UFT for patients with stage IV gastric carcinoma. *Am. J. Surg.*, **160**, 242.

MAEHARA, Y., MORIGUCHI, S., SAKAGUCHI, Y. & 4 others (1990*c*). Adjuvant chemotherapy enhances long-term survival of patients with advanced gastric cancer following curative resection. *J. Surg. Oncol.*, **45**, 169.

OTA, K., TAGUCHI, T. & KIMURA, K. (1988). Report on nationwide pooled data and cohort investigation in UFT phase II study. *Cancer Chemother. Pharmacol.*, **22**, 333.

SCHABEL, F.M. (1975). Concepts for systemic treatment of micrometastases. *Cancer*, **35**, 15.

SEIFERT, P., BAKER, L.H., REED, M.L. & VAITKEVICIUS, V.K. (1975). Comparison of continuously infused 5-fluorouracil with bolus injection in treatment of patients with colorectal adenocarcinoma. *Cancer*, **36**, 123.

TSUKAGOSHI, S., HASHIMOTO, Y., FUJII, G., KOBAYASHI, H., NOMOTO, K. & ORITA, K. (1984). Krestin (PSK). *Cancer Treat. Rev.*, **11**, 131.

UCHIDA, A. & HOSHINO, T. (1980). Clinical studies on cell-mediated immunity in patients with malignant disease. I. Effect of immunotherapy with OK-432 on lymphocyte subpopulation and phytomitogen responsiveness *in vitro*. *Cancer*, **45**, 476.